# SOX9 regulates epithelial‐mesenchymal transformation by mediating the Wnt/*β*‐catenin signaling pathway in hypospadias

**DOI:** 10.1002/pdi3.94

**Published:** 2024-07-09

**Authors:** Xueyu He, Zhicheng Zhang, Zhenmin Liu, Qiang Zhang, Chunlan Long, Lianju Shen, Guanghui Wei, Xing Liu

**Affiliations:** ^1^ Department of Urology Children's Hospital of Chongqing Medical University Chongqing China; ^2^ National Clinical Research Center for Child Health and Disorders Ministry of Education Key Laboratory of Child Development and Disorders Children's Hospital of Chongqing Medical University Chongqing China; ^3^ Chongqing Key Laboratory of Children Urogenital Development and Tissue Engineering Chongqing Key Laboratory of Pediatrics Children's Hospital of Chongqing Medical University Chongqing China; ^4^ China International Science and Technology Cooperation Base of Child Development and Critical Disorders Children's Hospital of Chongqing Medical University Chongqing China

**Keywords:** epithelial‐mesenchymal transformation, hypospadias, SOX9, WNT/*β*‐catenin

## Abstract

The transcription factor SOX9 is crucial in the development and differentiation of various tissues and cells. However, the roles of SOX9‐dependent genes and pathways in normal urethral development and the mechanism of hypospadias are unclear. This study collected 15 foreskin tissue specimens from patients who underwent hypospadias repair surgery and compared them to normal foreskin tissue specimens obtained during circumcision. The expression levels of SOX9, WNT signaling pathway markers, and epithelial‐mesenchymal transition (EMT) markers were analyzed in both groups. It was found that mRNA and protein levels of SOX9, WNT signaling pathway, and EMT mesenchymal markers were significantly reduced in the hypospadias group compared to the normal foreskin group. In contrast, mRNA and protein levels of epithelial markers were significantly increased in the hypospadias group. Immunofluorescence confirmed the decrease in SOX9 expression. Experiments using siRNA to inhibit SOX9 expression in foreskin fibroblasts yielded similar results to the hypospadias group. The findings suggest that down‐regulation of SOX9 expression may contribute to the development of hypospadias by down‐regulating the WNT pathway and inhibiting EMT. These findings provide new insights into the embryonic development of the urethra.

## Graphical abstract

The inhibition of SOX9 in mesenchymal cell may mediates the aberrant expression of the Wnt/*β*‐catenin signaling pathway EMT process, leading to the development of urethral hypospadias.

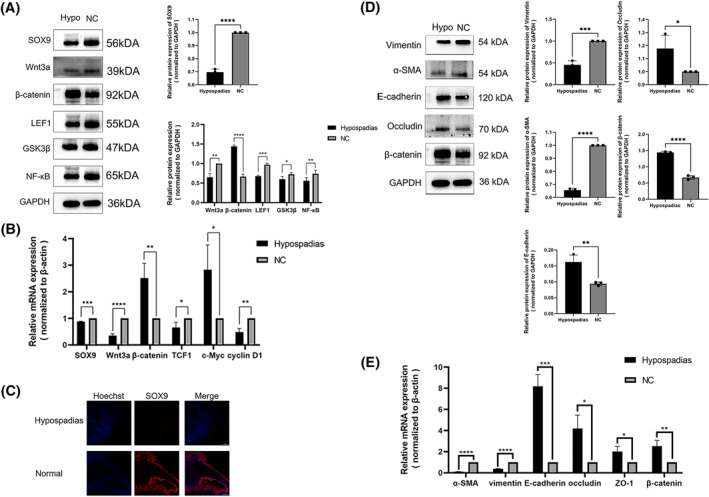

## 1 INTRODUCTION

Hypospadias is a prevalent congenital external genital malformation among male children. Its incidence slightly varies across countries and regions, ranging from 0.52% to 3.42%, and has been increasing annually.^1^ The clinical indications of hypospadias consist of an ectopic urethral orifice, an absence of ventral foreskin, excessive dorsal foreskin, and ventral curvature of the penis.^2^ Numerous theories regarding genetic predisposition and hormonal influences leading to hypospadias have been proposed.^3^ Currently, hypospadias may negatively impact the mental health of children and their future fertility. Surgery is considered the sole viable treatment option, however, it is prone to multiple complications.^4^ It is therefore crucial to acquire a comprehensive understanding of the etiology of hypospadias to effectively prevent and treat this condition.

SOX9 belongs to the family of transcription factors known as sox (Sry‐related high mobility group box). It is identified by a high mobility group (HMG) DNA‐binding domain and is involved in embryogenesis.^5^ SOX9 is pivotal in mediating embryonic development and sex determination.^6^ Reports suggest that mutations in SOX9 are responsible for hypospadias.^7^ Discovering fresh molecular mechanisms of SOX9 can enhance the comprehension of SOX9's pathogenesis in hypospadias, which holds immense significance.

The Wingless‐related integration site (Wnt)/β‐catenin signaling pathway is a conserved pathway regulating cell movement, polarity, proliferation and differentiation.^8^ Mutations in this pathway have been associated with human birth defects, cancer and other illnesses.^9^, ^10^ SOX9 is intriguingly known to upregulate *β*‐catenin expression in the Wnt pathway and thus influence downstream effectors to promote cancer advancement.^11^

There is ample evidence indicating that the excessive expression of SOX9 triggers the activation of the WNT signaling pathway, regulating the advancement of lung, gastric, rectal, and human prostate cancers.^12^, ^13^, ^14^ These cancers all prompt epithelial‐mesenchymal transition (EMT), a crucial biological process wherein epithelial cells transform into mesenchymal cells for tissue generation or regeneration.^15^, ^16^ There is ample evidence indicating that the excessive expression of SOX9 triggers the activation of the WNT signaling pathway, regulating the advancement of lung, gastric, rectal, and human prostate cancers.^12^, ^13^, ^14^ Additionally, EMT plays a significant role in urethra development. As shown in our previous research, EMT transforms epithelial cells into mesenchymal cells during urethral seam fusion, leading to hypospadias if blocked.^17^, ^18^, ^19^

**Aim**: However, little is known about the combined role of SOX9, WNT/β‐catenin signaling and EMT in the progression of hypospadias and there are no related reports. Therefore, this study aims to investigate the mechanism of SOX9 in hypospadias to fill this gap.

## 2 MATERIALS AND METHODS

### 2.1 Acquisition of hypospadias tissues

Foreskin specimens were acquired from children who received treatment for hypospadias repair and circumcision at the Children's Hospital of Chongqing Medical University. Patients with endocrine abnormalities, cryptorchidism, or hermaphroditism were excluded. The study consisted of 10 circumcised patients (with a mean age of 5 years) and 15 hypospadias patients (mean age of 3.5 years). Prior to the procedure, a written informed consent was obtained from both children and their guardians. This study was approved by the Ethics Committee of the Children's Hospital at Chongqing Medical University.

### 2.2 Reverse transcription‐polymerase chain reaction (RT‐PCR)

After either storing tissues at −80°C or transfecting cells for 48 h, the total RNA was isolated using the TRIzol (Invitrogen, Carlsbad) procedure. The isolated RNA was then reverse transcribed for mRNA analysis, following the instructions given by RT Master Mix for qPCR II (HY‐K0511A, MCE, Monmouth Junction). Subsequently, the reaction was conducted using SYBR Green qPCR Master Mix (HY‐K0523, MCE). The RT‐PCR program was carried out as follows: 5 min at 95°C, followed by 40 cycles consisting of 10 s at 95°C and 30 s at 60°C. Tsingke Biotechnology Co. Ltd (Beijing) synthesized the specific primers for SOX9, Wnt3a, *β*‐catenin, TCF1, cyclin D1, Vimentin, *α*‐SMA, E‐cadherin, ZO‐1, Occludin and *β*‐actin (refer to Table 1). *β*‐actin was used as the internal reference, and the expression data were calculated using the 2−ΔΔCt method. The tests were repeated three times.

**TABLE 1:** Primers Sequences and SOX9 siRNA Sequences.

Gene primers	Sequence (5ʹ−3ʹ)
SOX9	Forward: GACAGCCCCCTATCGACTTC
	Reverse: CAAACTCGTTGACATCGAAGG
Wnt3a	Forward: TGGGATGGTGTCTCGGGAGTTC
	Reverse: TCGTTGTTGTGGCGGTTCATGG
*β*‐catenin	Forward: TGGATTGATTCGAAATCTTGCC
	Reverse: GAACAAGCAACTGAACTAGTCG
TCF1	Forward: ACCGCAACCTGAAGACACAAGC
	Reverse: GCAATGACCTTGGCTCTCATCTCC
c‐Myc	Forward: AGCAGCGACTCTGAGGAGGAAC
	Reverse: TCCAGCAGAAGGTGATCCAGACTC
Cyclin D1	Forward: GCCCTCGGTGTCCTACTTCAAATG
	Reverse: TCCTCCTCGCACTTCTGTTCCTC
Vimentin	Forward: AGGCAAAGCAGGAGTCCACTGA
	Reverse: ATCTGGGCGTTCCAGGGACTCAT
*α*‐SMA	Forward: CTGCTGAGCGTGAGATTGTC
	Reverse: CTCAAGGGAGGATGAGGATG
E‐cadherin	Forward: AGCCCCGCCTTATGATTCTCTG
	Reverse: TGCCCCATTCGTTCAAGTAGTCAT
Occludin	Forward: AACTTCGCCTGTGGATGACTTCAG
	Reverse: TTTGACCTTCCTGCTCTTCCCTTTG
ZO‐1	Forward: GCTACGCTATTGAATGTCCCTG
	Reverse: CGACCAGAATGATCTGATGCC
*β*‐actin	Forward: AGTTGCGTTACACCCTTTCTT
	Reverse: CACCTTCACCGTTCCAGTTTT
SiRNA	Sequences (5ʹ−3ʹ)
SOX9	Forward: CGCUCACAGUACGACUACATT
	Forward: UGUAGUCGUACUGUGAGCGTT

### 2.3 Western blotting assay

The protein extraction process involved dissolving cells or tissue in a mixture of RIPA buffer (HY‐101032, MCE) and PMSF (HY‐B0496, MCE) in a ratio of 100:1. Following protein measurement using the bicinchoninic acid method, the protein samples were added to 10% SDS‐PAGE gels for electrophoresis. Afterward, 5% nonfat milk was used to block the PVDF membranes (Millipore, Billerica) for 1 h before incubating with primary antibodies targeting various antigens. SOX9 (ab185966, Abcam), Wnt3a (A0642, Abclonal Biotechnology Co. Ltd), *β*‐Catenin (A19657, Abclonal), GSK3β (R22868 ZEN‐BIO), LEF1 (382664, ZEN‐BIO), NF‐κB (R25149, ZEN‐BIO), Vimentin (R22775, ZEN‐BIO), *α*‐SMA (A17910, Abclonal), E‐Cadherin (ab76055, Abcam), ZO‐1 (21773‐1‐AP, Proteintech, Chicago, IL, USA), Occludin (sc‐8144, Santa Cruz Biotechnology, Santa Cruz, CA, USA) and total GAPDH (R24404, ZEN‐BIO) were utilized in this study. The membranes underwent incubation with goat anti‐rabbit (511203, ZEN‐BIO), goat anti‐mouse (511103, ZEN‐BIO), and rabbit anti‐goat (550094, ZEN‐BIO) secondary antibodies. Signals were detected with the use of the Bio‐Rad Imaging Laboratory System (Bio‐Rad). The band density of the experimental results was evaluated through the Image Lab software, and the expression level of the target protein was determined by the band density value of the target protein/internal reference protein band density value. The experiments were conducted thrice.

### 2.4 Cell culture and transfection

Obtaining foreskin specimens from normal children after circumcision with informed consent of family members. The foreskin fibroblasts were isolated using the established extraction method, followed by primary culture purification, and the cell culture was conducted according to previously published procedures. The fibroblast cells were cultured in a mixture of F12/DMEM (GIBCO, Burlington, ONT) supplemented with 10% fetal bovine serum (GIBCO) and 1% penicillin/streptomycin (Beyotime Biotechnology Co. Ltd). The cells were incubated at 37°C in a CO_2_ incubator with 5% CO_2_. Negative control (NC) siRNA and SOX9‐targeting siRNA (Table 1) were procured from TsingkeBio. The lipofection process was performed according to the Lipofectamine™2000 instructions (Thermo Fisher Scientific), with 1 × 10^4^ and 5 × 10^5^ cells seeded in each well of 48‐ and 6‐well culture plates, respectively. A serum‐free medium was provided for starvation treatment a day prior to transfection. The experimental and control groups were transfected with SOX9 siRNA and NC siRNA, correspondingly. Cells were gathered 48–72 h after transfection for future experiments.

### 2.5 Immunofluorescence

The foreskin tissues were fixed in 4% paraformaldehyde for 72 h, embedded in paraffin, and sliced into 4 *μ* m sections. After being baked in an oven at 37°C for 48 h, the sections required antigen retrieval with a citrate solution. The sections of foreskin tissue and the crawling sheets of foreskin fibroblasts after fixed with 4% paraformaldehyde were merged with 0.2% Triton X‐100 in PBS for 20 min and incubated with 0.5% BSA for 1 h. Primary antibodies against SOX9 (ab185966, Abcam), Wnt3a (A0642, Abclonal), *β*‐Catenin (A19657, Abclonal), and ZO‐1 (21773‐1‐AP, Proteintech) were used to incubate the slides overnight at 4°C in 5% BSA for an hour. The slides were then incubated for an additional hour at 25°C with the Cy3‐conjugated secondary antibody (CWBIO) before being analyzed with a fluorescence microscope (Nikon).

### 2.6 Statistical analysis

GraphPad Prism software version 9.0 (GraphPad Software, San Diego) was utilized for the analysis. Mean ± standard deviation (SD) was the expression used for the data. An independent *t*‐test was performed to compare the data of two groups with normal distribution.

## 3 RESULTS

### 3.1 Down‐regulation of SOX9 and Wnt signaling in hypospadias tissues

**FIGURE 1:**
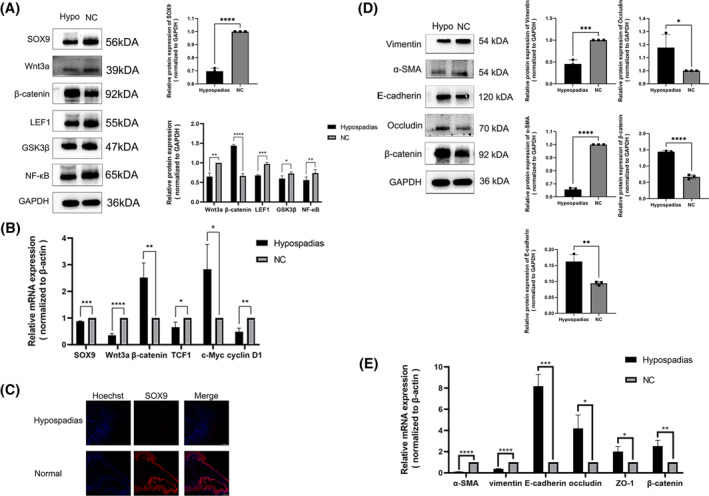
Relative expression of SOX9, Wnt/*β*‐catenin signaling pathway and epithelial‐mesenchymal transition markers in hypospadias foreskin tissues. (A) The protein expressions of SOX9, Wnt3a, *β*‐catenin, LEF1, GSK3β, NF‐κB were measured by Western blotting. (B) Detection of SOX9, Wnt3a, *β*‐catenin, TCF1, c‐Myc, cyclin D1 mRNA levels (*p < 0.05, **p < 0.01, ***p < 0.001, ****p < 0.0001). (C) Immunofluorescence showed that the expression of SOX9 in Hypospadias foreskin group was significantly lower than that in Normal foreskin group(fluorescence microscope × 200). (D) Western blotting analyses of *α*‐SMA, Vimentin, E‐cadherin, Occludin and *β*‐catenin protein levels of tissue specimens. (E) Detection of *α*‐SMA, Vimentin, E‐cadherin, Occludin and *β*‐catenin mRNA levels (*p < 0.05, **p < 0.01, ***p < 0.001, ****p < 0.0001).

Firstly, we examined the function of SOX9. Our analysis of single‐cell RNA sequencing data from the mouse genital tubercle indicated that SOX9 is specifically present in mesenchymal cells. We confirmed the presence and down‐regulation of SOX9 in hypospadias samples through RT‐PCR and Western Blot analysis. Furthermore, our immunofluorescence results highlighted significantly lower expression of SOX9 in the hypospadias foreskin group compared to the normal foreskin group. We utilized RT‐PCR and Western blot techniques to investigate the Wnt signaling pathway and observed a significant reduction in both mRNA and protein levels of SOX9, Wnt3a, LEF1, GSK3β, NF‐κB, TCF1, and cyclin D1 in the samples of hypospadias. Conversely, the levels of *β*‐catenin and c‐Myc were considerably increased (see Figure 1A,B,C).

### 3.2 Changes in EMT markers in hypospadias tissues

Next, we examined alterations in EMT markers. The study revealed that there was a decrease in the expression of mesenchymal markers Vimentin and *α*‐SMA in hypospadias tissues, whereas there was an increase in epithelial markers E‐cadherin, ZO‐1, *β*‐catenin, and Occludin (*p* < 0.05) as shown in Figure 1D,E. The decrease in mesenchymal markers and the increase in epithelial markers suggest that the EMT process is obstructed.

### 3.3 Transfection of SOX9 siRNA into foreskin fibroblast

It was investigated whether there is a connection between these markers and what role they play in their respective segments.

**FIGURE 2:**
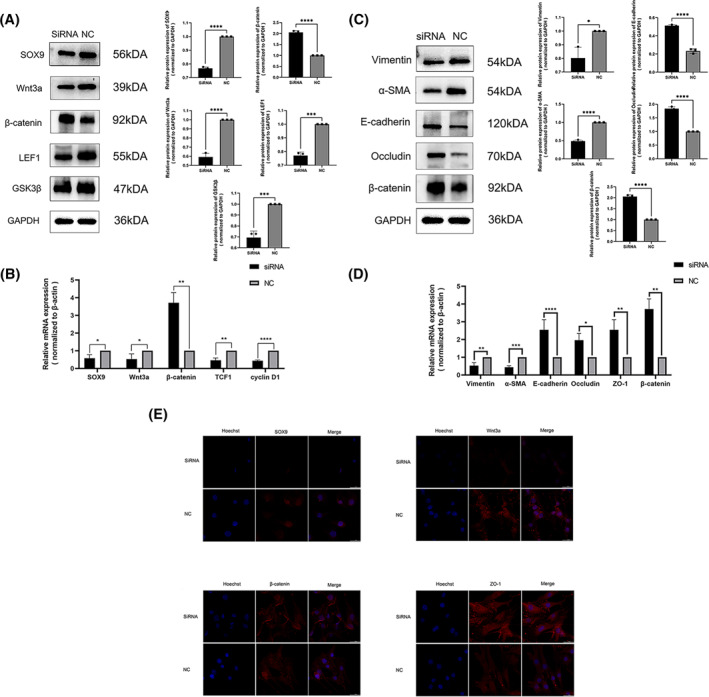
The Relative expression of SOX9, Wnt/β‐catenin signaling pathway and EMT markers in foreskin fibroblast cells transfected with SOX9 siRNA. Western blotting (A) and Western blotting RT‐PCR (B) analyses of SOX9, Wnt3a, *β*‐catenin, LEF1, GSK3β, TCF1, c‐Myc, cyclin D1 mRNA and protein levels in foreskin fibroblast cells from the SOX9 siRNA group compared with the normal control. Protein and mRNA levels of *α*‐SMA, Vimentin, E‐cadherin, Occludin and *β*‐catenin by Western blot (C) and RT‐PCR(D). (E)Expression of SOX9, Wnt3a, *β*‐catenin and ZO‐1 in SOX9‐transfected cells by immunofluorescence in the siRNA group and the NC group (fluorescence microscope, × 600). (*p < 0.05, **p < 0.01, ***p < 0.001, ****p < 0.0001).

To examine this hypothesis, we identified siRNA that effectively decreased SOX9 levels in foreskin fibroblasts. The effect of SOX9 siRNA on Wnt signaling pathway genes and downstream coding genes in foreskin fibroblasts was validated by RT‐PCR and Western blot analysis, which included Wnt3a, *β*‐catenin, GSK3β (Figure 2A,B). Additionally, we have confirmed that the expression of the LEF1 gene, which is regulated by *β*‐catenin, may be directly regulated by SOX9. This regulation was found to decrease when treated with siRNA.

The relative expression of Wnt signaling and EMT markers following SOX9 siRNA transfection in foreskin fibroblasts.

We decreased the expression of Wnt3a by using siRNA to downregulate SOX9 expression, providing evidence of its positive regulation. Simultaneously, the expression of mesenchymal markers Vimentin and *α*‐SMA related to EMT decreased, while the expressions of epithelial markers E‐cadherin, Occludin, ZO‐1, and *β*‐catenin increased (Figure 2C,D). These results resemble the expression seen in hypospadias tissues.

### 3.4 Immunofluorescence in siRNA group and NC group

The immunofluorescence staining results indicated a decrease in the expression of SOX9 and Wnt3a, whereas the expression of *β*‐catenin and ZO‐1 increased in the siRNA group (Figure 2E). These findings suggest that the downregulation of SOX9 affects EMT via the regulation of the Wnt signaling pathway.

## 4 DISCUSSION

Hypospadias is characterised by an atypical ventral opening of the urethra, as well as an abnormal ventral curvature of the penis and dorsal foreskin accumulation. It is believed that a combination of genetic and environmental factors contribute to the condition. However, the precise mechanism remains unknown. The abnormal expression of certain genes may disrupt the normal development of the urethra, leading to hypospadias.^17^ Academics have confirmed that the urethra forms through the gradual crimping and fusion of the urethral plate from the bottom to the tip, resulting in a urethral seam.^18^ This process involves the fusion of the urethral plate, the interaction of epithelial and mesenchymal cells, as well as the apoptosis of epithelial cells.^19^ Geller's research on hypospadias patients indicates that the most significant differences in hypospadias are found in mesenchymal stem cells, fibroblasts, and stromal cells.^20^ Malformations in the male urethral development, including hypospadias, are likely caused by the abnormal functions of these cells.^21^

SOX9 is involved in various signaling cascades, including the Wnt/β‐Catenin signaling pathway that plays a crucial role in reproductive development. Wnt/β‐Catenin signaling is essential for urethra development. The occurrence of severe hypospadias in both sexes among endodermal and ectodermal *β*‐catenin knockout animals indicates that the deregulation of any of these functions can potentially contribute to the development of congenital external genital defects in humans.^22^ *β*‐Catenin is essential in the ectoderm to uphold tissue integrity, likely via cell‐cell adhesion in GT outgrowth. The observation evokes thoughts of EMT, where *β*‐cetenin serves as one of its markers.

SOX‐Wnt/β‐catenin interactions have been demonstrated to regulate growth, development, and disease progression in various circumstances.^23^ SOX9 positively regulates several genes in the Wnt/β‐catenin signaling pathway.^24^ Overexpression of SOX9 stimulates the Wnt/β‐catenin signaling pathway, and the SOX9‐Wnt/β‐catenin axis governs the development of human lung, gastric, and rectal cancers.^24^, ^25^, ^26^, ^27^ EMT plays a key role in most cancers and is an important process in urethral embryogenesis. During male urethral development, the urethra forms through the gradual curling and fusion of urethral plates from proximal to distal, resulting in the urethral suture.^17^ This process involves the fusion of the urethral plate, interaction between epithelial and mesenchymal cells, and apoptosis of epithelial cells, which includes the EMT process.^19^, ^24^ The EMT process is considered a crucial step during the process of urethral plate fusion. During the formation of a complete urethra, some epithelial cells become encapsulated by mesenchymal cells and are unable to migrate. As a result, some of these cells undergo EMT and transform into mesenchymal‐type cells, which allows them to cross the basement membrane and move contralaterally to form the complete urethra.^28^, ^29^, ^30^ This process involves the loss of some epithelial cells through apoptosis. If the process is blocked, the epithelial cells of the urethral plate will prevent the migration of mesenchymal cells.^18^ This can result in abnormal fusion of the urethral sutures and impaired formation of the urethra, which may lead to hypospadias. Our previous work has demonstrated that EMT leads to the remodeling of epithelial cells in the urethral suture into mesenchymal cells after the fusion of the urethral plate.^31^, ^32^ Technical term abbreviations are explained when first used. It has been shown in studies that various signaling pathways, including TGF‐β, SHH, and Wnt, can mediate EMT.^33^ Knockout of SOX9 has been demonstrated to inhibit the proliferative EMT process of papillary thyroid cancer cells by inhibiting the Wnt/β‐catenin signaling pathway.^34^ Consequently, it is speculated that the SOX9‐Wnt/β‐catenin signaling pathway may exert a significant influence on EMT.

To investigate the roles of SOX9, WNT/β‐catenin signaling and EMT in hypospadias progression, we analyzed foreskin tissues obtained from children with hypospadias. Compared with normal foreskin, we observed reduced expression of SOX9, Wnt/β‐catenin pathway signaling and mesenchymal markers Vimentin and *α*‐SMA in hypospadias tissue. In contrast, levels of epithelial markers E‐cadherin, Occludin, ZO‐1 and *β*‐catenin were increased. The loss of E‐cadherin expression is a crucial indicator of EMT. However, the heightened expression of epithelial markers in hypospadias tissue indicates that EMT is suppressed. This pattern aligns with our prior research on hypospadias model rats and bolsters our conclusion.

We utilised siRNA to hinder the expression of SOX9 in human foreskin fibroblasts and assessed the alterations of these markers. We discovered that the alterations were akin to those seen in hypospadias tissues. Additionally, these findings were similar to our earlier studies on DEHP's impact on hypospadias in rodents.^24^ We therefore postulate that insufficient expression of SOX9 results in the development of hypospadias by lessening the Wnt signaling pathway, which consequently leads to anomalous EMT.

## 5 CONCLUSION

Our study is the pioneering work to reveal that the knockdown of SOX9 enhances the expression of epithelial markers in EMT by reducing the markers of the Wnt/*β*‐catenin signaling pathway. These results are in line with our clinical data and show promising prospects for treating hypospadias. However, we did not carry out any experiments to restore the expression of SOX9 and Wnt signaling pathways, which would have aided in confirming their roles. Consequently, more experiments are necessary to fill these knowledge gaps.

## AUTHOR CONTRIBUTION

**Xing Liu**, **Zhicheng Zhang** and **Guanghui Wei**: conceived and designed the experiments; **Xueyu He**: performed the experiments; **Xueyu He**: analyzed the data; **Chunlan Long** and **Lianju Shen**: contributed reagents/materials/analysis tools; **Xueyu He**: wrote the paper; **Zhicheng Zhang**, **Zhenmin Liu** and **Qiang Zhang**: assisted to perform the experiment. All authors contribute to the article and approved the final manuscript.

## CONFLICT OF INTEREST STATEMENT

No conflict of interest is reported by the authors.

## ETHICS STATEMENT

This study was authorized by the Ethics Committee of Children's Hospital of Chongqing Medical University (license numbers:2022‐0318). Written informed consents were obtained from both children and their guardians.

## Data Availability

The data that support the findings of this study are available from the corresponding author upon reasonable request.
